# Perceptual Processing Speed, Social Intelligence and Football Refereeing Performance: The Conditional Role of Attentional Control

**DOI:** 10.3390/jintelligence14040058

**Published:** 2026-04-01

**Authors:** Pedro Teques

**Affiliations:** 1 Department of Sports Science, Exercise and Health, School of Life Sciences and Environment, University of Trás-os-Montes and Alto Douro, 5000-801 Vila Real, Portugal; pteques@utad.pt; 2 Research Center in Sports Sciences, Health Sciences and Human Development—CIDESD, 5000-801 Vila Real, Portugal; 3 Portugal Football School, Portuguese Football Federation, 1495-733 Oeiras, Portugal

**Keywords:** applied intelligence, expert decision making, cognitive control, sport officiating, referee performance

## Abstract

Football refereeing involves rapid decision-making in dynamic, uncertain, and socially demanding environments. This study examined an integrative cognitive-behavioral model of football refereeing performance, focusing on perceptual processing speed (PPS), attentional control (AC), and social intelligence (SI). Sixty-one male football referees (*M*_age_ = 30.04, *SD* = 4.06) enrolled in a national talent development program across multiple competitive seasons participated in the study. At the beginning of each season, referees completed standardized, ability-based assessments of PPS (processing speed task), AC (selective and inhibitory task), and SI (performance-based social intelligence measure). Refereeing performance was operationalized using season-standardized end-of-season officiating ratings assigned by the national refereeing authority. Mediation analyses did not support AC or SI as mechanisms transmitting the effect of PPS on performance. However, moderation analyses revealed a significant PPS × AC interaction, indicating that attentional control amplified the positive association between perceptual processing speed and refereeing performance. PPS emerged as a robust predictor of performance, particularly among referees with high attentional control. Social intelligence showed a positive bivariate association with performance but did not function as a mediator or moderator in multivariate models. These findings support an interactive and ecological view of applied intelligence in football refereeing, emphasizing functional coordination highlighting the functional coordination of cognitive resources rather than isolated cognitive abilities as key to performance under real-world competitive demands.

## 1. Introduction

The role of football officials has undergone a profound metamorphosis (Webb et al., 2025). No longer merely an arbiter of disciplinary boundaries, the modern elite referee functions as a high-stakes performance manager, a real-time information processor, and a social regulator within a complex and dynamic system (Pietraszewski et al., 2014). Each match presents a cascade of approximately 130–150 active decisions (Helsen & Bultynck, 2004), each requiring the integration of partial, ambiguous visual data, the application of complex, contingent laws, and the navigation of intense socio-emotional pressures. All these decisions are made under intense psychological stress and public scrutiny (Pizzera et al., 2022), rendering the football pitch a particularly rich ecological context for the study of applied human cognition.

Paradoxically, the frameworks for selecting and training referee performance remain anchored in an outdated paradigm. An overwhelming emphasis persists on physical conditioning (e.g., repeated sprint ability, VO_2_ max) and declarative knowledge of the Laws of the Game (Helsen et al., 2024). Whilst undeniably important, this approach neglects the core psychosocial mechanisms that determine the quality and accuracy of the decision-making process itself. Although previous research has examined individual cognitive skills in officiating contexts, integrative and empirically tested models explaining how multiple cognitive and psychosocial resources interact to support refereeing performance remain scarce. In particular, it remains unclear whether higher-order executive and socio-cognitive processes operate as mechanisms or boundary conditions through which perceptual efficiency translates into performance outcomes under ecologically valid conditions.

The present investigation seeks to address this critical gap by proposing and testing an integrative cognitive-behavior model of officiating performance. It posits that three distinct yet interacting psychosocial constructs form the essential pillars of officiating expertise: (1) Perceptual processing speed (PPS) (Salthouse, 2000); (2) Attentional control (AC), which is the capacity for sustained, focused attention and resistance to interference (Eysenck et al., 2007); and (3) Social intelligence (SI), such as the ability to perceive, reason about, and manage social interactions and dynamics (Guilford, 1967). The ultimate criterion is real-world in football refereeing, including an end-of-season rating that corresponds to a composite metric derived from expert assessments of technical accuracy, tactical positioning, game management, and authority.

### 1.1. Perceptual Processing Speed

Traditional cognitive-psychometric analysis, focused on isolated mental components, offers a limited view of refereeing expertise (Helsen et al., 2023). To truly understand officiating excellence, a theoretical perspective is needed that recognizes the situated and emergent nature of decision-making. Ecological dynamics provides this framework, conceptualizing cognition as a property of the coupled performer-environment system (Araújo et al., 2025). Through this lens, Perceptual Processing Speed (PPS), measured by instruments (e.g., WAIS coding subtests), undergoes a fundamental reconceptualization. It ceases to be interpreted merely as an internal cognitive skill and instead becomes a measurable index of the referee’s capacity to attune to informational invariants within the perceptual-motor workspace.

In ecological psychology, perception exists for action (Gibson, 1979). A referee does not process “raw” visual data to later construct an abstract mental representation of a technical decision. Instead, they are immersed in a field of affordances (i.e., opportunities for action, such as calling a foul or playing advantage) that are specified by higher-order relational variables in the optic array (Mangalam et al., 2024). An offside, therefore, is not a calculated geometric fact but a directly perceived relational state: the invariant spatial relationship between attacker, defender, and the ball at the exact moment of the pass (Araújo & Kirlik, 2008). Expertise lies in the rapid and reliable detection of these relevant informational patterns amidst the chaotic flow of the game.

Thus, a high score on PPS tests reflects much more than efficient neural transmission. It reflects an embodied perceptual exploration strategy, such as a heightened sensitivity to task-specific informational patterns and greater effectiveness in calibrating visual search (Teques et al., 2017). The referee with high PPS can quickly direct their focus to the most informative areas (e.g., the last defender’s line, the passer’s foot), resisting capture by salient but irrelevant events (e.g., a falling player, a protest). This is not passive filtering but active, intelligent sampling of the environment. This attunement allows them to pick up the dynamic relational approach to the game, such as how player trajectories converge or the space between them shrinks, which specifies the future likelihood of a foul or a scoring opportunity, enabling prospective control of officiating action.

Therefore, PPS, measured psychometrically, becomes a proxy for the functional efficacy of the perception-action cycle within the referee-environment system. The referee must soft-assemble functional behaviors, including positioning, signaling, or communicating, that are adapted to the immediate, evolving constraints of the environment (Araújo et al., 2025).

### 1.2. Attentional Control

The ecological perspective, which frames the referee as a system attuned to action-specifying information, inherently demands a robust executive mechanism to safeguard that attunement (Linson et al., 2018). This brings us to the relevance of attentional control (AC). While perceptual processing speed (PPS) provides the bandwidth for perceptual intake, AC governs the fidelity and stability of that process over time. In the demanding ecology of a football match, the referee’s perceptual system is under constant siege from distractors, both external and internal (Samuel et al., 2024). Externally, the environment is replete with irrelevant yet salient information, including the roar of a partisan crowd or orchestrated player appeals designed to influence decisions (Teques, 2025). These stimuli are not mere background noise; they actively compete for limited cognitive resources. Internally, distractors are equally consequential and include physiological feedback from fatigue or anxiety regarding performance consequences (Samuel et al., 2024).

Consequently, officiating expertise transcends perceiving the action; it relies on the active and prospective maintenance of task-relevant focus on the primary perceptual scene, including the potential infractor and the kinematic cues that specify an impending foul, while simultaneously inhibiting both external intrusions and internal cognitive interference (van Biemen et al., 2018). This executive function is grounded in sustained selective attention and resilient inhibitory control across prolonged periods of performance. Research on cognitive fatigue in sustained decision-making tasks demonstrates that depletion of top-down attentional resources leads to attentional narrowing and increased omission errors, such as failures to detect critical events (van der Linden et al., 2003). In refereeing contexts, this may manifest as missing peripheral infractions during late-game set pieces, when cognitive resources are most compromised.

Accordingly, high levels of attentional control reflect more than momentary attentional acuity. They represent attentional endurance, that is, the capacity of the executive control system to preserve the integrity of the perception-action cycle under sustained pressure and cognitive fatigue (Tanaka et al., 2014). From a theoretical standpoint, attentional control may therefore assume more than one functional role within the regulation of football refereeing performance, supporting both mediation and moderation perspectives (Draheim et al., 2022; Samuel et al., 2024). On the one hand, it may operate as a supportive mechanism, facilitating the stabilization of perceptual processing over time and thereby contributing indirectly to performance by sustaining perceptual-decisional efficiency. On the other hand, attentional control may function as a regulatory constraint, delimiting the conditions under which perceptual processing speed can be effectively expressed in real-world officiating contexts. In this latter role, attentional control does not transmit perceptual efficiency but instead determines the extent to which perceptual speed can be translated into adaptive performance under ecologically valid demands.

### 1.3. Social Intelligence

While the perceptual and attentional systems provide the foundational machinery for officiating, the domain of application is fundamentally a dynamic social system. Football refereeing is a complex social context governed by formal laws but enacted through volatile human emotions and interpersonal interactions (Teques, 2025). Consequently, a complete model of officiating expertise must account for a discrete dimension of intellect focused on this behavioral realm. Drawing upon Guilford’s (1967) structure of intellect model, we posit Social Intelligence (SI) as the cognition of behavioral content, a distinct operational faculty encompassing the abilities to perceive, comprehend, and evaluate psychological states and interactions, and to generate adaptive behavioral responses. For a referee, this manifests in the continuous, real-time application of three core functions derived from Guilford’s framework: the perception of behavioral relations (e.g., detecting escalating tension or deceptive simulation), the evaluation of broader behavioral systems (e.g., gauging team volatility or crowd influence), and the production of behavioral transformations (e.g., generating communicative acts to deescalate conflict).

During a football game, the referee must navigate a landscape of social affordances (Birk & Manning, 2024), where player behaviors present constant opportunities for either conflict escalation or effective management. The task extends beyond judging actions to managing the social perception of procedural justice. Successful management, often through calibrated non-verbal communication such as deliberate eye contact, controlled gesturing, and intentional proxemics (Teques, 2025), can prevent minor incidents from cascading into major disciplinary events, thereby preserving match control and flow. This behavioral cognition may represent a potential interface between perceptual judgments and their social acceptance.

Although perceptual processing speed, attentional control, and social intelligence have often been examined separately, fewer studies have considered how these processes operate jointly in shaping refereeing performance. From an applied cognition perspective, performance in dynamic officiating environments likely depends on how attentional resources are regulated and integrated with social-interaction demands (Helsen et al., 2023). Accordingly, the present study examines both the direct and conditional relationships among these variables, testing whether attentional control and social intelligence shape the strength of the association between perceptual processing speed and refereeing performance.

### 1.4. The Purpose of This Study

**Figure 1:**
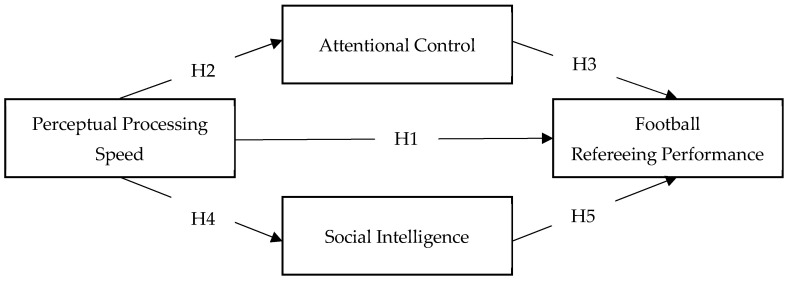
Hypothesized mediation model of perceptual processing speed and refereeing performance via social intelligence and attentional control.

The general objective of this study is to examine the cognitive-behavioral processes that distinguish effective football officiating. Drawing on applied and ecological perspectives, the study tests an integrative model including perceptual processing speed (PPS), attentional control (AC), and social intelligence (SI) as key contributors to real-world refereeing performance. Specifically, it is hypothesized that it will be positively associated with refereeing performance (H1). It is further expected that PPS will be positively associated with AC (H2) and AC with refereeing performance (H3). Additionally, PPS will be positively associated with SI (H4) and SI will be related with refereeing performance (H5) (see Figure 1). Considering these associations, it is expected that SI and AC will mediate the relationship between PPS and refereeing performance (H6 and H7, respectively).

**Figure 2:**
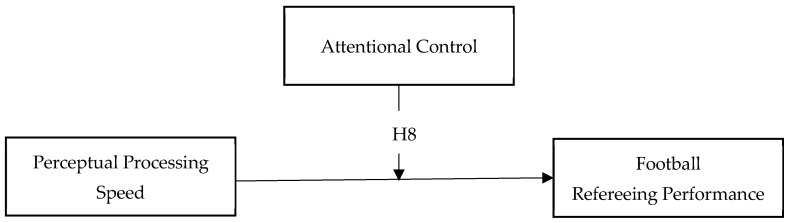
Hypothesized moderation effect of attentional control on the association between perceptual processing speed and refereeing performance.

Beyond these associations, the study examines AC as a potential moderating factor within the refereeing performance system. Accordingly, it is hypothesized that AC will condition the relationship between PPS and refereeing performance (H8), such that the positive association between PPS and performance will be stronger at higher levels of AC. This hypothesis reflects the assumption that perceptual efficiency alone is insufficient for optimal football refereeing and must be supported by sustained executive regulation to be effectively expressed under ecologically valid performance demands (see Figure 2). Given that data were collected across multiple competitive seasons, the proposed models also incorporated competitive season as a covariate to examine its unique contribution on the relationships between variables.

## 2. Methods

### 2.1. Study Design

This study adopted a cross-sectional, correlational design aimed at testing a multiple mediation model examining the indirect effects of attentional control and social intelligence on the relationship between perceptual processing speed and refereeing performance.

### 2.2. Participants and Procedures

The sample comprised 61 male football referees participating in the Refereeing Academy program. Data were collected across multiple cohorts between 2018 and 2025. Participants ranged in age from 26 to 36 years (*M* = 30.04, *SD* = 4.06). Regarding educational background, 26% of the referees had completed secondary education (12th grade), 51% held a bachelor’s degree, and 23% had completed a master’s degree. Refereeing experience ranged between 10 and 18 years (*M* = 14.52 years, *SD* = 5.01). All participants were actively officiating at national-level competitions at the time of data collection and were enrolled in a formal talent identification and development pathway aimed at progression to professional football competitive categories.

The study was approved by the Ethics Committee of the Portugal Football School—Portuguese Football Federation (Approval No. 11/2021, approved in 11 April 2022). Ethical approval was obtained specifically for the use of routinely collected institutional data for research purposes. Each referee was evaluated at the beginning of each competitive season as a part of a national talent identification and selection program, the Refereeing Academy, promoted by the Refereeing Committee. This program is designed to identify and prepare referees with high potential to progress to the second national category of football officiating. The Academy consists of an intensive one-week training and assessment period, during which referees participate in a structured set of activities, including theoretical evaluations, practical on-field assessments, and psychological assessment. The cognitive and psychosocial measures included in the present study were administered within this framework, under standardized conditions, as part of the psychological evaluation component of the program. All participants provided written informed consent prior to participation. All assessments were conducted individually by the author of the study, following standardized administration procedures. The psychological assessment required approximately 45 min to 60 min per participant, including task instructions and transitions between measures.

### 2.3. Measures

To capture the cognitive-behavioral associations underlying officiating performance, a multi-method assessment battery was employed, integrating standardized performance-based cognitive tests, social-cognitive functioning, and ecologically valid indicators of real-world refereeing performance. Raw scores were retained for the cognitive measures to preserve sensitivity to individual differences in processing efficiency within a relatively homogeneous high-performance cohort. This approach prioritizes functional variability relevant for regression- and interaction-based analyses over normative classification. All instruments selected have robust psychometric support in the literature and are widely used in applied cognitive and performance research.

#### 2.3.1. Perceptual Processing Speed

Perceptual Processing Speed was assessed using two standardized subtests from the Wechsler Adult Intelligence Scale IV (Wechsler, 2008), namely Coding and Symbol Search, which together constitute the core indicators of the Processing Speed Index. These tasks are designed to evaluate the efficiency with which individuals can rapidly perceive, discriminate, and respond to visual information under strict time constraints, while maintaining accuracy.

- The Coding subtest requires participants to copy symbols that are paired with numbers according to a provided key, as quickly and accurately as possible within a fixed time limit. Successful performance relies on rapid visual scanning, associative learning, sustained attention, and fine motor coordination. In applied terms, Coding captures the individual’s capacity to maintain a fast and stable perception–action loop, integrating perceptual input with motor execution while minimizing errors. In the context of football officiating, this subtest is interpreted as a proxy for the referee’s ability to rapidly encode and act upon visually specified information during continuous play, where decisions must be executed fluently and without hesitation.
- The Symbol Search subtest assesses visual discrimination speed and attentional efficiency by requiring participants to determine whether one of two target symbols appears within a group of visually similar symbols. The task emphasizes rapid perceptual comparison, selective attention, and resistance to interference from non-target stimuli, with minimal motor demands relative to Coding. From an ecological perspective, Symbol Search reflects the referee’s ability to swiftly detect task-relevant visual invariants within a cluttered perceptual field, such as identifying critical spatial relationships between players while ignoring irrelevant or misleading cues.

Both subtests were administered individually following standardized WAIS procedures. Raw scores were derived from the number of correct responses completed within the allotted time for each subtest. Consistent with prior research on processing speed, the two subtests were treated as complementary indicators of PPS, capturing both visuomotor efficiency (Coding) and perceptual discrimination speed (Symbol Search). Higher scores reflect faster and more accurate perceptual processing. The WAIS IV coding and symbol search subtests demonstrate reliability and validity across adult populations, supporting their use as robust indicators of individual differences in perceptual processing speed in applied performance contexts (Arias, 2025).

#### 2.3.2. Attentional Control

Attentional Control was assessed using the d2-R Test of Attention (Brickenkamp et al., 2010), a revised and internationally validated version of the original d2 test, specifically designed to measure concentrated attention and inhibitory control under time pressure. The d2-R is a paper-and-pencil cancellation task that places high demands on sustained selective attention, visual scanning efficiency, and response inhibition, making it particularly suitable for assessing attentional functioning in applied performance contexts.

The test consists of 14 rows of characters, each containing a sequence of visually similar stimuli (the letters “d” and “p”) marked with one to four small dashes arranged above and/or below each letter. Participants are instructed to scan each row from left to right and to mark only the target stimuli, defined as the letter “d” with exactly two dashes, while ignoring all non-target stimuli (e.g., “d” with one, three, or four dashes, and all “p” stimuli). Each row is processed under a strict time limit of 20 s, requiring rapid discrimination and continuous attentional engagement.

Performance on the d2-R yields several indices that reflect different attentional components. In the present study, the primary indicator of Attentional Control was Concentration Performance, calculated as the total number of correctly marked target stimuli minus commission errors (incorrect markings). This index captures the efficiency of attentional focus by integrating processing speed with accuracy and inhibitory control. Higher concentration performance scores indicate a more effective ability to maintain task-relevant attention while suppressing impulsive or erroneous responses. In addition, omission errors (missed targets) and commission errors were examined descriptively to provide complementary information regarding attentional lapses and inhibitory failures.

#### 2.3.3. Social Intelligence

Social Intelligence was measured using the Four-Factor Test of Social Intelligence, originally developed by O’Sullivan and Guilford (1976). This ability-based test is designed to capture core socio-cognitive processes that underlie an individual’s capacity to understand, interpret, and anticipate social interactions. The Four-Factor Test comprises four performance-based subtests, each tapping a distinct socio-cognitive ability that contributes to effective social functioning:

- Cartoon Predictions. Participants are presented with a socially ambiguous cartoon scenario and are required to select, from multiple response options, the most plausible continuation of the interaction. This subtest assesses the ability to infer intentions, anticipate behavioral outcomes, and reason prospectively about social situations based on limited contextual information.
- Expression Grouping. Participants categorize facial expressions or social gestures according to shared emotional or interpersonal meaning. This task emphasizes the perceptual discrimination and categorization of non-verbal social cues, skills directly relevant to detecting emotional states and early signs of social escalation in competitive match contexts.
- Social Translations. This subtest presents brief interpersonal statements or dialogues, requiring participants to identify the interpretation that best captures the implicit meaning or communicative intent. It indexes the ability to decode indirect messages and social nuance, which is central to understanding players’ appeals, protests, and strategic communication during matches.
- Missing Cartoons. Participants view a sequence of cartoon-based social actions with one critical frame omitted and must select the image that logically completes the interaction. Successful performance reflects the ability to infer continuity in social behavior and anticipate subsequent relational developments.

Raw scores on the Guilford-O’Sullivan Four-Factor Test of Social Intelligence were obtained by summing correct responses (1 = correct; 0 = incorrect) across the four performance-based subtests (i.e., Cartoon Predictions, Expression Grouping, Social Translations, and Missing Cartoons), yielding a total composite score. Similar to Myznikov et al. (2021), total raw scores were converted into a five-level standardized scale (1–5), with higher values indicating greater social intelligence. In the present study, raw scores from all subtests were summed to create a composite index of social intelligence, with higher scores reflecting superior socio-cognitive proficiency.

#### 2.3.4. Referee Performance

Refereeing performance was operationalized using the official end-of-season performance rating assigned to each referee by the national refereeing authority. This rating represents a cumulative evaluation based on match-by-match assessments conducted throughout the competitive season by independent external observers appointed by the Refereeing Committee. Observers are certified officials trained in standardized evaluation procedures.

Match performance evaluations follow a structured observation framework that integrates both quantitative ratings and qualitative analysis across multiple performance domains. Specifically, referees are assessed on: (a) technical and disciplinary decision-making and overall game management; (b) physical condition, movement patterns, positioning, and proximity to play; (c) communication and coordination with the officiating team, including assistant referees and VAR when applicable; and (d) overall performance appraisal, including contextual judgment of critical incidents and consistency across the match. These domains are evaluated using standardized criteria and rating scales, complemented by written qualitative feedback documenting key incidents and officiating decisions.

Individual match evaluations are aggregated across the season to generate a final end-of-season performance rating expressed on a standardized numerical scale. As referees included in the sample were evaluated across different competitive seasons, direct comparison of raw end-of-season scores could be affected by contextual variability between seasons, such as differences in rating distributions or evaluative stringency. To address this issue while preserving ecological validity, performance scores were standardized within each season prior to analysis. Specifically, each referee’s final season rating was transformed into a z-score relative to the mean and standard deviation of ratings within the corresponding season. Higher values indicate superior officiating performance relative to peers within the same season. These standardized performance scores were used as the outcome variable in all subsequent analyses.

### 2.4. Data Analysis

Data was analyzed using IBM SPSS Statistics (version 31). Prior to hypothesis testing, data screening procedures were conducted. Descriptive statistics (i.e., means, standard deviations, ranges) were computed for all study variables, and bivariate associations were examined using Pearson correlations. Assumptions relevant to regression-based mediation were evaluated, including inspection of univariate outliers, linearity, and multicollinearity (VIF < 5; Hair et al., 2022). There was no missing data, as all ability-based tests were administered individually.

The hypothesized parallel multiple mediation model was tested using PROCESS Model 4 (Hayes, 2022), estimating the direct effect of PPS on refereeing performance (c′ path), as well as the indirect effects through AC and SI (a1b1 and a2b2 paths, respectively). In addition, the total effect of PPS on performance (c path) was computed. Indirect effects were evaluated using bias-corrected bootstrap confidence intervals based on 5000 resamples, as recommended for mediation analysis due to the typically non-normal sampling distribution of indirect effects (Tibbe & Montoya, 2022). An indirect effect was considered statistically significant when the 95% bootstrap confidence interval did not include zero.

To examine whether attentional control conditioned the relationship between perceptual processing speed and refereeing performance, moderation analyses were conducted using PROCESS Model 1. Perceptual processing speed was specified as the focal predictor (X), attentional control as the moderator (W), and refereeing performance as the outcome variable (Y). All predictors were mean-centered prior to the computation of interaction terms. Given that data were collected across multiple competitive seasons, additional sensitivity analyses were performed to examine the robustness of the mediation and moderation models. Specifically, season was included as a covariate to account for potential temporal clustering effects. Effect sizes were reported using unstandardized coefficients with standard errors and confidence intervals, and model fit was summarized using the proportion of explained variance (*R*^2^) for each regression equation.

## 3. Results

### 3.1. Preliminary Analyses

Preliminary analyses were conducted to examine the distributional properties of the study variables and their bivariate associations prior to the main regression-based models. Descriptive statistics for perceptual processing speed (PPS), social intelligence (SI), attentional control (AC), and refereeing performance, based on raw scores, are presented in Table 1 for descriptive and contextual interpretation. However, because referees were evaluated across different competitive seasons, all regression-based mediation and moderation analyses were conducted using season-standardized z-scores of performance to ensure comparability across seasons. Multicollinearity diagnostics were conducted to assess the stability of the regression estimates. Variance inflation factors (VIF) ranged from 1.64 to 3.12, and tolerance values exceeded 0.30 for all predictors, indicating acceptable levels of collinearity. Inspection of condition indices and variance proportions revealed no evidence of problematic multicollinearity.

Bivariate correlations revealed significant positive associations among all study variables. PPS was strongly correlated with attentional control (*r* = 0.81, *p* < .001) and moderately correlated with social intelligence (*r* = 0.59, *p* < .001). PPS was also strongly associated with refereeing performance (*r* = 0.64, *p* < .001). Social intelligence showed moderate positive correlations with both attentional control (*r* = 0.60, *p* < .001) and refereeing performance (*r* = 0.49, *p* < .001). Attentional control was moderately associated with performance (*r* = 0.42, *p* = .002).

### 3.2. Mediation Analysis

**Figure 3:**
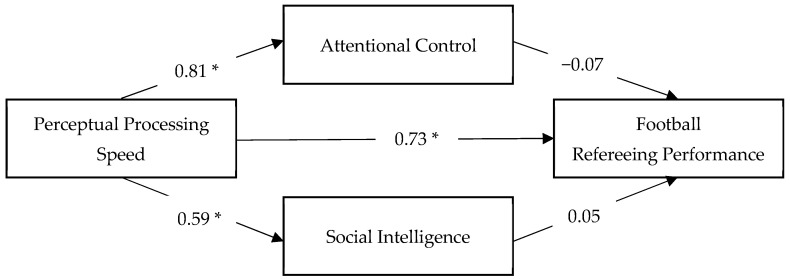
Mediation model linking perceptual processing speed to football refereeing performance via attentional control and social intelligence. *Note*. Standardized paths are presented between variables. * *p* ≤ 0.05.

The relationships among perceptual processing speed (PPS), attentional control (CI), social intelligence (SI), and refereeing performance were further examined using a mediation framework. Specifically, using the PROCESS macro for SPSS (Model 4; Hayes, 2022), PPS was specified as the independent variable, attentional control and social intelligence were entered simultaneously as mediators, and refereeing performance served as the dependent variable. Findings of the proposed mediation model analysis are displayed in Figure 3 and summarized in Table 2 (direct effects) and Table 3 (indirect effects).

Results showed that PPS was a significant predictor of both proposed mediators. PPS was positively associated with social intelligence, accounting for 34.6% of the variance in SI. Similarly, PPS was associated with attentional control, explaining 65.7% of its variance. When attentional control and social intelligence were simultaneously entered into the model, PPS remained a significant predictor of refereeing performance. In contrast, neither social intelligence nor attentional control reached conventional levels of statistical significance, although both exhibited trend-level associations with performance. The full model accounted for 46.6% of the variance in refereeing performance (see Table 2).

Bootstrap analyses with 5000 resamples further indicated that the total indirect effect of PPS on performance through attentional control and social intelligence was not statistically significant (indirect effect = −0.12, BootSE = 0.11, 95% CI [−0.31, 0.12]). Examination of specific indirect effects revealed that neither the pathway through social intelligence (indirect effect = 0.11, 95% CI [−0.01, 0.26]) nor the pathway through attentional control (indirect effect = −0.23, 95% CI [−0.42, 0.04]) was statistically significant, as all confidence intervals included zero. The contrast between the two indirect effects was also non-significant (contrast = 0.35, 95% CI [−0.00, 0.62]) (see Table 3).

Taken together, these findings indicate that, contrary to the initial mediation hypothesis, attentional control and social intelligence did not function as mediating mechanisms in the relationship between perceptual processing speed and refereeing performance. Notably, attentional control displayed a negative coefficient in the full model, suggesting that its role may not be linear or transmissive. This pattern implies that attentional control may instead operate as a boundary condition influencing how perceptual processing speed is translated into performance outcomes, thereby motivating further examination of attentional control as a moderating variable.

### 3.3. Moderation Analysis

To further clarify the functional role of attentional control in the relationship between perceptual processing speed and refereeing performance, a moderation analysis was conducted. Grounded in theoretical perspectives that emphasize the interactive coordination of cognitive resources in dynamic decision-making environments, attentional control was conceptualized as a boundary condition shaping the translation of perceptual efficiency into performance. Accordingly, using the PROCESS macro for SPSS (Model 1; Hayes, 2022), perceptual processing speed (PPS) was specified as the independent variable, attentional control (AC) as the moderator, and refereeing performance as the dependent variable. All coefficients reported below are based on standardized (z-score) performance values.

**Figure 4:**
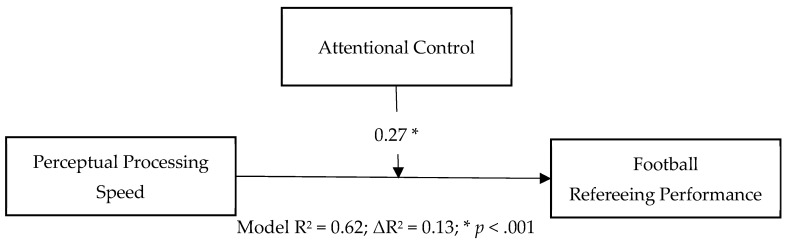
Moderation model testing attentional control as a moderator of the association between perceptual processing speed and football refereeing performance. Values shown represent the interaction effect (PPS × AC) and incremental variance explained (ΔR^2^).

The overall moderation model (Figure 4) was statistically significant and explained a substantial proportion of variance in refereeing performance, *R*^2^ = 0.624, *F*(4, 56) = 23.38, *p* < .001. PPS showed a positive main effect on performance (*β* = 0.84, SE = 0.12, *t* = 6.99, *p* < .001), whereas attentional control exhibited a significant negative main effect (*β* = −0.62, SE = 0.17, *t* = −3.73, *p* < .001). Crucially, the PPS × AC interaction was statistically significant (*β* = 0.27, SE = 0.06, *t* = 4.40, *p* < .001), indicating that the strength of the association between PPS and refereeing performance varied as a function of attentional control. The interaction term accounted for an additional Δ*R*^2^ = 0.130 of explained variance (*F*(1, 56) = 19.38, *p* < .001). The covariate season was not significant (*p* = .818).

**Figure 5:**
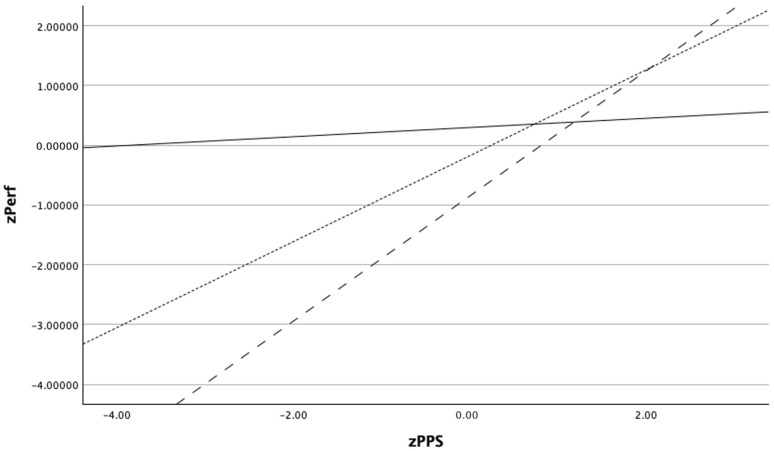
Simple slopes illustrating the moderating effect of attentional control (AC) on the relationship between perceptual processing speed (zPPS) and refereeing performance (zPerf). The positive association between zPPS and performance becomes progressively stronger at higher levels of AC (−1 SD, mean, +1 SD). *Note*. *Dashed line = High AC; solid line = Low AC; dotted line = Average AC*.

Conditional effects analyses showed that perceptual processing speed (PPS) was positively associated with refereeing performance at all examined levels of attentional control. As illustrated in Figure 5, the strength of this association increased progressively with higher levels of attentional control. Specifically, PPS exhibited a moderate positive effect on performance at low levels of attentional control (−1 SD; *β* = 0.60, SE = 0.13, *t* = 4.60, *p* = .001), a stronger effect at mean levels of attentional control (*β* = 0.87, SE = 0.12, *t* = 7.24, *p* < .001), and the strongest effect at high levels of attentional control (+1 SD; *β* = 1.15, SE = 0.14, *t* = 8.11, *p* < .001). This pattern indicates an amplifying moderation effect, such that higher attentional control is associated with a steeper positive relationship between perceptual processing speed and refereeing performance.

### 3.4. Covariate Analysis

Given that data were collected across multiple competitive seasons, additional analyses were conducted to examine whether competitive season influenced the mediators or refereeing performance, or altered the substantive pattern of results. Competitive season was entered as a covariate in mediation and moderation models.

Competitive season did not significantly predict attentional control, social intelligence, or refereeing performance (*p* > 0.05). Importantly, the direct effect of perceptual processing speed on refereeing performance remained statistically significant when season was included as a covariate. Likewise, the interaction between perceptual processing speed and attentional control remained significant, indicating that the mediation or moderation effect was not attributable to between-season variability.

## 4. Discussion

The present study examined how perceptual processing speed, attentional control, and social intelligence jointly relate to football refereeing performance. Overall, the findings provide partial support for the proposed model and offer novel insights into the interactive nature of cognitive resources underpinning football refereeing excellence. The first hypothesis examined whether perceptual processing speed represents a core cognitive foundation of refereeing performance. Across bivariate and multivariate analyses, PPS emerged as a predictor of end-of-season performance ratings, indicating that referees who process perceptual information more rapidly tend to perform better within their competitive context. This finding is consistent with research showing that expert performance in dynamic, time-pressured domains is characterized by superior perceptual-cognitive efficiency (Ericsson & Lehmann, 1996; Seidel-Marzi et al., 2024; Shangguan & Zha, 2025).

Within the ecological perspective adopted in this study, PPS reflects the efficiency with which it refers to action-specifying information embedded in the game environment (Gibson, 1979; Mangalam et al., 2024). In officiating, this involves the rapid detection of relational patterns, such as player trajectories and ball-body interactions, that specify affordances for action. The present findings therefore support ecological models of expertise that emphasize perception-action coupling as the primary mechanism underpinning skilled performance in sport (Araújo et al., 2019; Raab & Araújo, 2019).

Building on the central role of PPS, it was expected that attentional control would mediate the relationship between perceptual processing speed and refereeing performance. Contrary to expectations, this hypothesis was not supported. Although PPS was strongly associated with attentional control, and PPS exerted a significant total effect on performance, attentional control did not transmit this effect in the mediation model. The findings align with contemporary perspectives suggesting that, in complex environments, cognitive resources tend to operate in parallel and in an interactive manner (e.g., Pessoa, 2023; Maren, 2022; Wu & Molnár, 2022). In this sense, attentional control delineates the task constraints under which perceptual efficiency is successfully translated into functional performance.

The proposed model hypothesized whether social intelligence would mediate the relationship between PPS and refereeing performance. Although social intelligence was positively associated with refereeing performance at the bivariate level, it did not emerge as a significant mediator or moderator in the multivariate models. This pattern may reflect the fact that social intelligence, as originally conceptualized, captures domain-specific abilities related to interpersonal understanding and behavioral regulation (Guilford, 1967; O’Sullivan & Guilford, 1976). In officiating contexts, such abilities may be more closely linked to broader aspects of match management, communication, and perceived procedural justice (Teques, 2025). Consequently, the contribution of social intelligence may become more salient under specific situational demands, including conflict escalation or heightened social pressure, which were not explicitly modeled in the present study.

Beyond the absence of a mediating effect, the observed pattern of results indicated that attentional control may play a conditional role in shaping how perceptual processing speed translates into performance. Guided by this interpretation, attentional control was subsequently examined as a moderator of the PPS-performance relationship. Consistent with this hypothesis, moderation analyses demonstrated that the positive association between perceptual processing speed and football refereeing performance was progressively amplified as levels of attentional control increased.

Inspection of the simple slope pattern further clarifies the nature of this moderating effect. Although perceptual processing speed was positively associated with refereeing performance across all levels of attentional control, the strength of this association increased systematically as attentional control increased (Figure 5). This pattern indicates that rapid perceptual processing confers a greater performance advantage when referees are able to sustain attentional focus under task demands, whereas lower attentional control constrains the functional expression of perceptual efficiency. Importantly, controlling for the covariate did not meaningfully alter the direction or magnitude of the main and interaction effects, supporting the robustness of the observed relationships.

This interaction pattern helps clarify the initially counterintuitive negative coefficient observed for attentional control in the mediation model. The results suggest that attentional control functions as a boundary condition that determines the extent to which perceptual processing speed can be effectively expressed under performance constraints. When attentional control is low, the benefits of rapid perceptual processing are attenuated, likely due to increased susceptibility to distraction, emotional interference, or cognitive fatigue (Draheim et al., 2022). Conversely, higher levels of attentional control may help maintain task-relevant perceptual engagement over time (Klostermann, 2020), enabling referees to sustain functional coupling with critical informational cues over extended periods of play. However, when considered in isolation, elevated attentional control may reflect a more effortful and cautious processing style that is suboptimal under conditions of extreme time pressure and uncertainty. In such contexts, excessive top-down control can constrain adaptive visual exploration and delay action selection (Grüner et al., 2023), particularly when perceptual processing speed is insufficient to support rapid information uptake.

### 4.1. Implications for Theory

The present findings contribute to theoretical discussions on applied cognition in sport by highlighting the coordinated contribution of multiple cognitive resources to football refereeing performance. The results indicate that refereeing effectiveness reflects the functional interplay between perceptual processing speed and attentional control under performance constraints, moving beyond linear or stage-based interpretations of cognitive functioning. Perceptual processing speed appears to provide the cognitive bandwidth required to engage with rapidly evolving game information, while attentional control conditions the extent to which this perceptual efficiency can be sustained and expressed over time and under pressure (Sun et al., 2026).

From a theoretical standpoint, these findings also inform the relationship between ecological perspectives on cognition and the use of standardized cognitive measures. Although the instruments employed are decontextualized and originate from information-processing traditions, they can be interpreted as indexing general cognitive resources whose functional expression emerges within specific performance environments. Crucially, the outcome variable in the present study was derived from the official football refereeing performance evaluation system, based on real-world expert assessments conducted across competitive seasons. This ecologically valid performance criterion provides a meaningful bridge between laboratory-based cognitive indicators and situated performance, supporting the interpretation that basic cognitive resources can predict effectiveness when their expression is constrained and shaped by authentic environmental demands (e.g., Araújo et al., 2025). In this context, attentional control does not operate as an intermediate mechanism through which perceptual efficiency exerts its effects, but as a regulatory factor shaping when and how perceptual resources become behaviorally relevant in football refereeing contexts.

Social intelligence, while theoretically central to the social ecology of refereeing (e.g., Legg et al., 2025), appears to operate at a different level of regulation. Its contribution may be more closely related to interpersonal dynamics, communication, and match management than to core perceptual-decisional processes examined in the present models. Taken together, these findings support an integrative theoretical view in which football refereeing performance reflects the coordinated contribution of distinct cognitive resources, whose functional relevance depends on task demands and contextual constraints (e.g., Helsen et al., 2023), while allowing psychometric indicators to inform ecological interpretations without conflating measurement context with performance expression. This interpretation should also be considered in light of the psychometric limitations of standardized social intelligence measures. Ability-based tests assess social reasoning under decontextualized and low-pressure conditions and may therefore underestimate the contribution of interpersonal skills enacted under emotional load, time pressure, and social confrontation (Woods & Patterson, 2023). As such, caution is warranted when extrapolating performance on psychometric measures of social intelligence to complex, real-world officiating behavior.

### 4.2. Implications for Practice

From an applied perspective, these findings carry important implications for referee selection, development, and training. Assessment protocols that rely on isolated cognitive tests may fail to capture critical interaction effects between perceptual and attentional resources that underpin real-world officiating performance. Selection frameworks may therefore benefit from evaluating how these capacities co-function under realistic performance constraints. Similarly, development programs that simultaneously target perceptual attunement and attentional regulation, particularly under conditions of fatigue, pressure, and distraction, may yield greater performance gains (e.g., Pompa et al., 2025).

In training contexts, the results suggest that interventions should move beyond generic cognitive drills and instead embed perceptual and attentional challenges within football-specific scenarios. Examples include video-based decision-making tasks combined with concurrent attentional load (e.g., time pressure, secondary monitoring demands), on-field simulation exercises performed under physical fatigue, or role-playing training formats that require referees to maintain attentional focus while managing social and environmental distractors. Such approaches may better support the functional transfer of cognitive skills to competitive performance than isolated training of perceptual speed or attentional control alone.

### 4.3. Limitations and Future Directions

Several limitations of the present study warrant careful consideration and point toward directions for future research. First, the cross-sectional design precludes strong causal inference regarding the developmental or functional ordering of perceptual, attentional, and performance-related processes. Even though the tested models were theoretically grounded, it remains unclear whether higher perceptual processing speed facilitates the development of attentional control over time, whether sustained attentional demands shape perceptual efficiency, or whether both are co-shaped through prolonged exposure to elite officiating environments. Longitudinal designs tracking referees across seasons, or experimental studies manipulating perceptual or attentional load, would be particularly valuable in disentangling these reciprocal influences (Eayrs et al., 2024).

Second, although the sample size limits sensitivity to small interaction effects, the observed moderation effect was of moderate-to-large magnitude (ΔR^2^ = 0.15; f^2^ = 0.38). A post-hoc power analysis indicated that the study had sufficient power to detect an effect of this size. Nevertheless, replication in larger samples is warranted to further assess the stability and generalizability of interaction effects in football refereeing contexts.

Third, while the use of ecologically valid end-of-season performance ratings strengthens the applied relevance of the findings, such evaluations inevitably integrate multiple dimensions of officiating (e.g., technical accuracy, positioning, game management) and may be influenced by contextual or institutional factors. Future research could complement global performance ratings with more fine-grained behavioral indicators, such as decision accuracy under varying temporal constraints, positioning efficiency, or communication behaviors during critical match events. In this context, examining how cognitive fatigue and sustained vigilance affect the perception of affordances and the functional deployment of perceptual-attentional resources may further refine the mapping between cognitive processes and performance outcomes.

Fourth, the exclusive focus on male referees drawn from a talent identification and promotion program limits the generalizability of the results. Cognitive demands and developmental trajectories may differ across competitive levels, officiating roles, and gender. Expanding this line of research to include female referees, assistant referees, and officials operating in different competitive ecosystems would help determine the extent to which the present pattern of associations reflects a generalizable organization of cognitive resources in football refereeing or a context-specific configuration associated with male referees in high-performance pathways.

Finally, although the present study was grounded in ecological models of cognition and relied on an ecologically valid performance criterion, social intelligence was operationalized using a psychometric measure of generalized social reasoning. Such measures may capture latent cognitive resources whose functional expression depends on situational constraints and may therefore underestimate the role of social intelligence under conditions of heightened social or emotional complexity (e.g., conflict escalation or sustained player dissent). Moreover, the standardized ordinal scaling of the instrument may have reduced score variability and statistical power. Future research should therefore integrate situational indicators, alternative measurement approaches, and within-match variability to better capture when and how social intelligence becomes functionally relevant in football refereeing.

## 5. Conclusions

This study contributes to the literature on cognitive determinants of football refereeing performance by showing that perceptual processing speed constitutes a robust individual correlate of end-of-season performance ratings, while attentional control shapes the conditions under which this perceptual efficiency is functionally expressed. Contrary to transmission-based assumptions, higher-order cognitive and socio-cognitive resources did not mediate the relationship between perceptual processing speed and performance. Instead, attentional control operated as a conditional factor, amplifying the performance relevance of perceptual speed under shared variance. These findings support an interactional view of cognitive functioning in football refereeing, in which performance reflects the coordinated contribution of multiple cognitive resources rather than linear causal pathways. While the cross-sectional design and measurement constraints warrant cautious interpretation, the present results underscore the importance of considering how perceptual and attentional processes jointly relate to performance outcomes in applied decision-making domains. Future research employing longitudinal designs, larger samples, and context-sensitive measures may further clarify how these cognitive resources are developed and deployed across competitive demands.

## Figures and Tables

**Table 1:** Descriptive statistics and correlations among study variables (*n* = 61).

Variable	*M*	*SD*	1	2	3	4
1. Perceptual Processing Speed (PPS)	105.56	12.68	—			
2. Social Intelligence (SI)	2.62	1.09	0.592 *	—		
3. Attentional Control (AC)	175.32	31.51	0.814 *	0.598 *	—	
4. Referee Performance	8.93	0.14	0.639 *	0.490 *	0.418 *	—

Note. *M* = mean; *SD* = standard deviation. PPS = perceptual processing speed (composite score); SI = social intelligence; AC = Attentional control; Referee Performance = end-of-season performance rating. Pearson correlation coefficients are presented below the diagonal. * *p* < .01 (two-tailed).

**Table 2:** Mediation model direct effects of perceptual processing speed and refereeing performance via social intelligence, attentional control and season (covariate).

	Outcome: SI			Outcome: AC			Outcome: Ref Performance		
Predictor	*B*	*SE*	*β*	*B*	*SE*	*β*	*B*	*SE*	*β*
Perceptual Processing Speed (PPS)	0.46 *	0.09	0.59	0.64 *	0.07	0.81	0.62 *	0.15	0.73
Social Intelligence (SI)							0.03	0.02	0.05
Attentional Control (AC)							−0.02	0.01	−0.07
Season (Covariate)	−0.09	0.10	−0.12	0.05	0.01	0.10	0.00	0.01	0.00

Note. *B* = Unstandardized coefficients; *SE* = Standard error; *β* = Standardized coefficients; * *p* < 0.001.

**Table 3:** Indirect effects of the perceptual processing speed and refereeing performance via social intelligence and attentional control.

Indirect Effect	*β*	*BootSE*	*95% CI*
PPS → SI → Perf	0.11	0.08	[−0.31, 0.12]
PPS → AC → Perf	−0.23	0.12	[−0.42, 0.04]

Note. PPS = Perceptual Processing Speed (PPS); SI = Social Intelligence; AC = Attentional control; perf = refereeing performance; β = Standardised indirect effect; BootSE = Bootstrap standard error; CI = Confidence interval.

## Data Availability

The data presented in this study are not publicly available due to ethical and confidentiality restrictions. The dataset includes sensitive information related to official performance evaluations of football referees conducted within an institutional talent development program. Anonymized data may be made available by the corresponding author upon reasonable request and with permission from the relevant governing body.
